# Imported malaria in a cosmopolitan European city: A mirror image of the world epidemiological situation

**DOI:** 10.1186/1475-2875-7-56

**Published:** 2008-04-08

**Authors:** Juan Pablo Millet, Patricia Garcia de Olalla, Paloma Carrillo-Santisteve, Joaquim Gascón, Begoña Treviño, José Muñoz, Jordi Gómez i Prat, Juan Cabezos, Anna González Cordón, Joan A Caylà

**Affiliations:** 1 Epidemiology Service, Public Health Agency of Barcelona, Pza Lesseps, 1, 08023 Barcelona, Spain; 2 CIBER de Epidemiología y Salud Publica (CIBERESP), Spain; 3 Hospital Clínic, International Health Center (CRESIB), IDIBAPS, University of Barcelona, Villarroel 170, 08036 Barcelona, Spain; 4 Tropical Medicine and International Health Unit, Primary Health Care Drassanes Center, Avda Drassanes 17-21, 08001 Barcelona, Spain

## Abstract

**Background:**

International travel and migration have been related with an increase of imported malaria cases. There has been considerable immigration to Barcelona from low-income countries (LIC) in recent years. The objective is to describe the epidemiology and to determine the trends of the disease in Barcelona.

**Methods:**

Analysis of the cases notified among city residents between 1989 and 2005. Patients were classified as: tourists, voluntary workers, resident immigrants (visiting friends and relatives, VFR) and recently arrived immigrants. An analysis was conducted using the chi^2 ^test and comparison of means. As a measure of association we calculated the Relative Risk (RR) and Odds Ratio (OR) with a Confidence Interval of 95% (CI) and carried out a trends analysis.

**Results:**

Of the total of 1,579 imported cases notified, 997 (63.1%) lived in Barcelona city, and 55.1% were male. The mean age of patients was 32.7 years. The incidence increased from 2.4 cases/100,000 in 1989 to 3.5 cases/100,000 in 2005 (RR 1.46 CI:1.36–1.55). This increase was not statistically significant (trends analysis, p = 0.36). In terms of reason for travelling, 40.7% were VFR, 33.6% tourists, 12.1% voluntary workers and 13.6% were recently arrived immigrants. The most frequent species found was *Plasmodium falciparum *(71.3%), mainly in visitors to Africa (OR = 2.3, CI = 1.7–3.2). The vast majority (82.2%) had had some contact with Africa (35.9% with Equatorial Guinea, a Spanish ex-colony) and 96.6% had not completed chemoprophylaxis. Six deaths were observed, all tourists who had travelled to Africa and not taken chemoprophylaxis (3.9% fatality rate).

**Conclusion:**

Over the period studied there is an increase in malaria incidence, however the trend is not statistically significant. Lack of chemoprophylaxis compliance and the association between Africa and *P. falciparum* are very clear in the imported cases. Most of the patients with malaria did not take chemoprophylaxis.

## Background

Malaria is an infectious disease caused by an intracellular protozoan parasite of the genus *Plasmodium *and is transmitted by the bite of the female *Anopheles *mosquito. It is endemic in more than 100 countries and mainly concentrated in the tropics and some subtropical areas. Transmission varies between regions and between urban zones and it is estimated that the population that lives in risk-zones will continue to increase [1-3]. Currently, there are still 350–500 million new cases and more than one million deaths per year [1,4-6], 90% of which occur in Africa. Malaria has been identified as the disease with the greatest impact on morbidity in the world, contributing to the obstruction of social and economical development in many countries [3-6].

Malaria was eradicated from Europe in the seventies. In Spain, the last autochthonous case was diagnosed in 1961 and the official certificate of eradication was obtained in 1964. All of the diagnosed cases are imported with the exception of a few particular forms of transmission of malaria in airports and transmission via blood, in transfusions and between intravenous drug users [7]. The increase of international travel and immigration from endemic zones has caused an increase in the number of recorded cases in the last few years. Between 13,000 and 16,000 cases are notified annually in Europe (about 400 of the cases are from Spain) [8], with a fatality rate of 2–3% [2]. In Spain, malaria is also a notifiable disease. In addition to the general observation of an increase in cases between 1981 and 1987 [7], an increase has also been reported by some health centres [9,10]. The objective of the present study is to determine the epidemiological characteristics and evolution of malaria during a 17-year period in a city, which has experienced massive immigration from low-income countries (LIC) in recent years.

## Methods

### Study population

All of the notifications of malaria between January, 1989 and December 2005 were reviewed by the Epidemiology Service of the Barcelona Public Health Agency. The study included confirmed cases of malaria among Barcelona residents notified during the period of the study. Cases of any episode indicative of malaria confirmed in a laboratory were also considered, once possible relapse from a previous episode had been ruled out.

### Epidemiological survey

After every notification, a nurse filled out a questionnaire which collected patients' personal information, and dates of first symptoms, diagnosis, hospital admission and discharge. The following information was also collected: endemic geographical areas visited within 30 days prior to onset of symptoms, or the last country visited if it exceeded 30 days (*Plasmodium vivax/Plasmodium ovale*), the reason for travel, and, in the case of an immigrant, if they already resided in Barcelona or had only recently arrived to the city. For analysis purposes, the immigrants' children were also considered immigrants, even if they were born in Barcelona. The information about completion of chemoprophylaxis treatment, and visited area according to the level of risk of multi-resistant *Plasmodium falciparum *transmission, as defined by the WHO [11] (Types I to IV: minimum to maximum level of recommendation for prevention), was also noted.

Subjects were classified, based on birthplace and usual place of residence, into one of four categories of traveller: "tourists" (reside in a non-malaria country and went to an endemic area for vacation or work), "volunteers" (born in a non-malaria country, but reside in an area with malaria), and immigrants, subdivided into "visiting friends and relatives (VFR)" (born in areas with malaria, or their children born in Barcelona, who reside in an area without malaria and travel to their country of origin to visit friends or family) [7,12] and "arriving immigrants". The epidemiological survey allowed for differentiation between VFR and those recently arriving immigrants.

### Laboratory

The most relevant microbiological data were the type of species detected and the method used for diagnosis. The diagnostic criterion included the microscope observation of parasites and identification of species on a Giemsa-stained thick and thin bloodfilm. When the morphological characteristics of parasites were indeterminate they were considered *Plasmodium sp.*

### Statistical analysis

The quantitative variables were described using mean and standard deviation (SD), or median and interquartile range (IR) if the data did not follow a normal distribution. The chi square test was used to compare qualitative variables, while ANOVA and the corresponding non-parametric tests were used to compare the quantitative variables. In looking for trends, the best straight line fit was determined and a chi square test of the trend was calculated. For measures of association between different groups, a bi-variate Odds Ratio (OR) and Relative Risk (RR) was calculated with confidence intervals of 95% (CI) and logistic regression was used at multivariate level to adjust for age and sex.

The rates of incidence per 100,000 inhabitants in one year were calculated using the average population in a five-year period (there were 1,503,884 inhabitants in Barcelona in the 2001 census) [13]. SPSS version 13.0 and EpiInfo version 6 programs were used for statistical analysis.

## Results

### Incidences and trends

**Figure 1:**
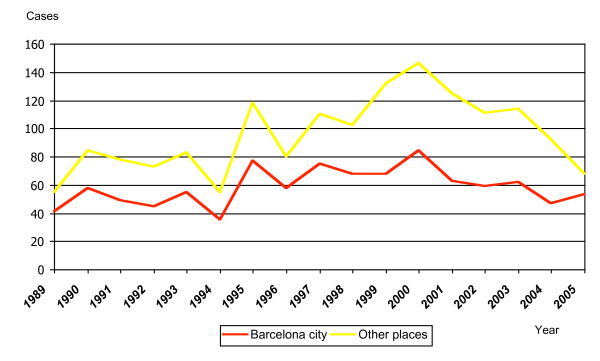
Evolution of notified malaria cases in Barcelona according to notification year and place of residence (1989–2005).

**Figure 2:**
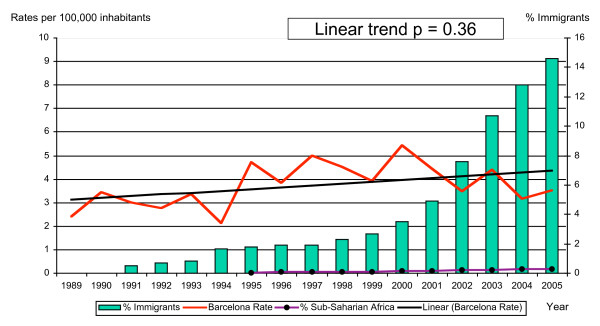
Comparison of malaria rates and percentage of immigrants among the general population of Barcelona (1989–2005).

The study included 1,579 cases, all imported except one, infected by transfusion. Barcelona city residents accounted for 997 cases (63.1%) (Figure 1). The incidence rates varied from 2.4/100,000 in 1989 to 3.5/100,000 in 2005, with a peak in 2000 (5.4/100,000) (Figure 2). The specific rates for the population born in Barcelona changed from 0.92 in 1991 to 1.47/100,000 in 2004 (RR = 1.6; CI: 1.48–1.71). The rates among VFR decreased from 64/100,000 in 1991 to 11.6/100,000 in 2004 (RR = 5.5; CI: 5.31–5.64) in the city. The trends analysis did not reveal any significant increase over the years (p = 0.36), even though the risk increased by almost 50% when comparing the years 1989 and 2005 (RR = 1.46; CI: 1.36–1.55). Thirty seven percent of the cases were notified in the months of September, October, and December.

### Group characteristics

**Figure 3:**
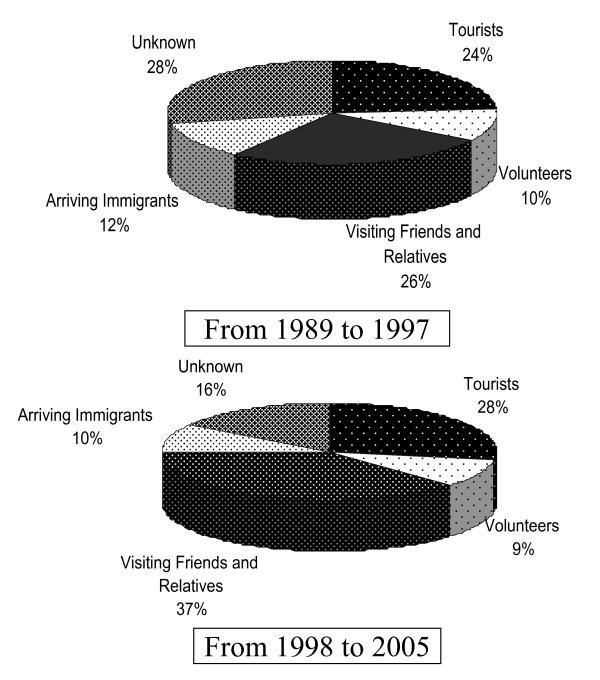
**Notified malaria cases in Barcelona by type of traveller 1989–2005. **Comparison of period of massive immigration (1998–2005) with the previous period (1989–1997).

The mean age was 32.7 years with SD of 15.7, and predominantly male (55.1%). Regarding the type of traveller, 40.7% were VFR, 33.6% tourists, 12.1% volunteers, and 13.6% recently arriving immigrants. A group could not be assigned in 215 cases (21.6%). Compared with tourists, the VFR were mainly young, had visited Africa, and did not complete chemoprophylaxis (p < 0.001) (Table 1). The annual trends in the number of cases by groups shows a great increase among tourists (Figure 3). It was also noted that 82.1% of the patients had travelled to an African country (35.9% to Equatorial Guinea).

**Table 1:** Epidemiological characteristics of the different groups of travelers affected by malaria in Barcelona (1989–2005).

	**Tourists N (%)**	**Volunteers N (%)**	**VFR N (%)**	**Recently AI N (%)**	**Total N (%)**	p-value*
Age (md, IR)	33 (27–41)	40.5 (31–57)	30 (21–39)	27 (24–41)	32.7 (15.7)	0.02
						
Sex						
Male	161 (61.2)	46 (48.4)	171 (53.9)	55 (51.9)	433 (55.1)	0.001
Female	102 (38.8)	49 (51.6)	146 (46.1)	51 (48.1)	348 (44.9)	
						
Duration of travel (md, IR)	30 (22–67)	88 (32–259)	31 (26–62)	-	31 (25–81)	0.81
						
Correct CP						
Yes	8 (4.5)	6 (10.4)	1 (0.4)	-	15 (2.7)	< 0.001
No	46 (25.8)	13 (22.4)	13 (5.3)	2 (2.9)	74 (13.4)	
Not taken	124 (69.7)	39 (67.2)	231 (94.3)	68 (97.1)	462 (83.8)	
Total	178 (32.3)	58 (10.5)	245 (44.5)	70 (12.7)	551 (100)	
						
Continent						
Asia	26 (10)	4 (4.2)	26 (8.2)	4 (3.8)	60 (7.7)	0.002
Africa	185 (70.6)	85 (89.5)	272 (85.8)	98 (92.4)	640 (82.1)	
America	49 (18.7)	6 (6.3)	19 (6)	4 (3.8)	78 (10)	
Oceania	2 (0.7)	-	-	-	2 (0.2)	
Total	262 (33.6)	95 (12.1)	317 (40.7)	106 (13.6)	780 (100)	

md.: median, IR: interquartile range, CP: chemoprophylaxis, VFR: visiting friends and relatives, AI: arriving immigrants.

* comparison between VFR and tourists.

The vast majority of cases (83.2%) came from the only two centres in Barcelona with Tropical Medicine Units, the Hospital Clinic and the Drassanes Primary Health Centre. Hospital admissions were required in 31.2% of cases, most being admitted to the Hospital Clinic (59%). The median length of stay was four days (IR 3–6). The median time from onset of symptoms until diagnosis was eight days (IR 5–20), nine days (5–23) among immigrants and 10 (6–25) among autochthonous patients (p = 0.35). *P. falciparum *infection was more prevalent in immigrants than autochthonous patients (OR = 2.1; CI: 1.6–2.9, p < 0.001), even though autochthonous patients were admitted more often than the immigrants (OR = 1.8; CI: 1.3–2.3, p < 0.001). One third of the immigrants (262, 33.5%), generally less severe cases, were treated at the Drassanes Primary Health Centre.

A total of 120 cases (12%) were children under 15; 86.7% were immigrants (OR = 2.6; CI: 1.4–4.8, p = 0.004), 91% came from Africa, and 1% from America (p < 0.001); 69.8% were infected with *P. falciparum*.

Standardized rates were calculated for each district in the city (data not shown). The highest annual incidence rate was in Ciutat Vella (District 1), followed by Nou Barris (District VIII) and Sarriá (District V) (rates of 10.1, 5.1, and 4.7/100,000, respectively). A significant association was observed between immigration and living in Nou Barris (OR = 5.2; CI: 3.1–8.6, p < 0.001) or in Ciutat Vella (OR = 2.3; CI: 1.5–3.3, p < 0.001) and between tourists and living in Sarriá (OR = 2.4; CI: 1.5–3.9, p < 0.001).

### Laboratory

**Figure 4:**
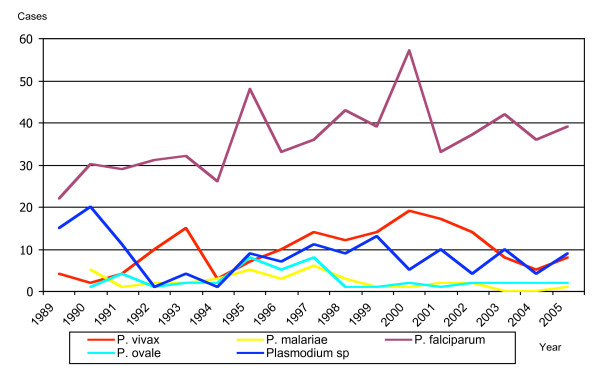
Distribution of isolated species in Barcelona city between 1989 and 2005 by notification year.

*Plasmodium falciparum *was the species most frequently identified, in 614 cases (71.3%) (Table 2). The smear did not reveal the species in 136 cases (13.6%), even though parasite was detected by the thick drop test. In terms of continents, *P. falciparum *was isolated most frequently from Africa (81.6%) (OR = 2.3; CI: 1.7–3.2, p < 0.001), while *P. vivax *was the most frequent in travellers from Asia (69.2%) (OR = 12.4; CI: 6.9–21.9, p < 0.001) and America (71.3%) (OR = 16.7; CI: 9.9–28.2, p < 0.001). Progression over time shows a clear prevalence of *P. falciparum *(Figure 4).

**Table 2:** Malaria in Barcelona: Plasmodium species by continent visited.

	**Africa**	**Asia**	**America**	**Oceania**	**Unknown**	**Total n (%)**
**P. falciparum**	565	16	22	0	11	614 (71.3)
**P. vivax**	55	45	62	2	2	166 (19.3)
**P. ovale**	41	1	2	0	0	44 (5.1)
**P. malariae**	31	3	1	0	2	37 (4.3)
**Plasmodium sp**	80	8	3	0	45	136 (13.6)
**Total**	772	73	90	2	60	997 (100)

### Chemoprophylaxis and lethality

Chemoprophylaxis was not taken or not completed correctly in 96.9% of the cases, while only 3.1% appeared to complete it correctly. Regarding the distribution of chemoprophylaxis by zone, 90% of the cases had visited a country with high risk of transmission of multi-resistant *P. falciparum *(Type IV Risk) (Table 3). There were significant differences between the groups of patients; tourists had the best adherence (p < 0.001), in spite of overall low compliance to chemoprophylaxis.

**Table 3:** Malaria in Barcelona 1989–2005: malaria risk and type of prevention (WHO, 2006) according to the different groups of patients.

Type of Prevention*	**Tourists**	**Volunteers**	**FVR**	**Recently AI**	**Total n(%)**
**I**	1	0	1	0	2 (0,3)
**II**	37	8	2	0	47 (6)
**III**	16	2	3	1	22 (2,8)
**IV**	206	81	312	105	704 (90)
**Unknown**	3	4	0	0	7 (0,9)
**Total n (%)**	263 (34)	95 (12)	318 (41)	106 (14)	782 (100)

VFR: visiting friends and relatives, AI: arriving immigrants.

* Depending on malaria risk in the area visited, the recommended prevention method may be mosquito bite prevention only (Type I), or mosquito bite prevention in combination with chemoprophylaxis (Type II to IV according to the *P. falciparum *presence and resistance in the area) [11].

Six deaths were observed, all attributed to *P. falciparum *(lethality rate of 1.05%) and all were tourists who had travelled to Africa (3.9% fatality rate), 66.7% being male. None had taken chemoprophylaxis. The average age of those who died was 51.3 years (SD 13.5) and was 32.5 years (SD 13.6) for those who did not (p = 0.003).

## Discussion

The increase in imported malaria cases from different countries is due to the augment of immigration from LIC and the number of people visiting endemic areas [5,6]. In Barcelona, despite a significant increase in the number of cases in the period 1981–1987 [7], and despite incidence being higher in 2005 than in the preceding five years, the trend observed in incidence was not statistically significant. Moderate increases have also been observed in Catalonia and Spain [14], in various European cities [15-18], the USA [19,20], and in Canada [21].

The rates of malaria observed in the autochthonous population were 1.5 times higher in 2004 than in 1991, probably due to increases in travel to endemic areas and failure to comply with chemoprophylaxis. On the other hand, the rate among the VFR group was more than five times higher in 1991 than in 2004. This is probably because the immigrant population registered in the 2004 census had grown, due to extensive immigration occurring in Spain, and the immigrants were primarily from Latin America and Northern Africa, areas with low incidences, or entirely free of malaria [13] (Figure 2). It is also possible that the sub-Saharan VFR group – lowest in number but highest in terms of risk of acquiring and importing malaria – do not yet go back to their countries as often due to the high cost of travel, precarious conditions and non-regularized residence status.

**Figure 5:**
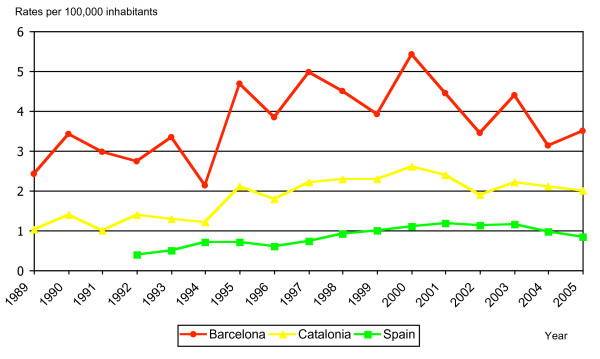
Evolution of malaria rates notified in Barcelona, Catalonia and Spain (1989–2005).

It is debatable if the impact of malaria at global level is underestimated [22,23]. In this study, which relies on the notifications from doctors, the number of cases could be underestimated, as observed in London and the Netherlands [23-25]. However, there has been good collaboration for many years between the Epidemiology Service and the two reference centres for imported diseases, where most of the cases in the city were visited and compiled. In fact, the difference in rates among Barcelona, Catalonia, and Spain (Figure 5) reflect not only the presence of high tourism activity and immigration in Barcelona, but also a better system of awareness, given the notable differences in incidence.

Immigrants from sub-Saharan Africa classified as VFR, followed by tourists, constitute the groups in Spain accounting for the highest numbers of cases [8,26,27], something also seen in various European cities [12,17,18]. As observed by other authors, these infected immigrants have probably acquired semi-immunity, thus their manifestation is less serious (less hospitalizations and there are no records of deaths), in contrast to the fatality observed among tourists who visit Africa (3.9% fatality rate) [28]. The characteristics of the immigrant and autochthonous subjects are very different. Whereas the immigrants, many having migrated for economic reasons, normally go to visit their families in areas with hyperendemic malaria, in low-income countries, the other subjects are tourists who, when they travel, stay for shorter periods, visit areas with less endemic malaria, and take more precautions (have less risk of mosquito bites through staying in air-conditioned hotels, and adhere better to chemoprophylaxis). However, one of the limitations of the study was that twenty two percent of the patients could not be assigned to a group because it was not known if they were recently arriving immigrants or if they already lived in Barcelona or, in the case of autochthonous patients, because the reason for their travel, whether as a tourist or for volunteer purposes, was not recorded.

Other countries with high immigration rates have also observed a large number of imported malaria cases among VFR [16]. These immigrants visit their homeland with their children, rarely or never exposed to the disease and consequently less likely to possess natural acquired immunity, and none of them take (or if they take it, do not complete) chemoprophylaxis. In Barcelona, where the immigration boom is recent, the VFR cases could increase in the future when immigrants and their children have the opportunity to visit their mother country. It is interesting to note that in this study the majority of the affected minors were children of immigrants from sub-Saharan Africa [10,27] and less of the affected minors were from families from South America, a region of lower malaria prevalence. It is also noteworthy that a great number of the cases are either immigrants from the Spanish ex-colony Equatorial Guinea or their children, as seen in the past [29]. Like many of the VFR, Equatorial Guinean subjects, as well as their children and other family members, continue to travel to their country without undertaking chemoprophylaxis. When they come back, despite being asymptomatic, they go for a check up at the Drassanes PHC and hence many cases were detected through screening. Indeed, if friends or relatives come to visit them from Equatorial Guinea, they sometimes take them to the doctor too. This could explain why so many cases of falciparum malaria present in the specialized health centre of the poorest district of the city. More studies are needed in order to better characterize this population at a high risk of importing malaria.

The most affected districts within the city are those that are most socio-economically disadvantaged and that experienced the most immigration (Nou Barris and Ciutat Vella) [13]. The district with the highest socio-economic level (Sarria) is the third most affected, probably due to the influence of tourists and volunteer workers. To decrease malaria among VFR and their children, it is fundamental to make chemoprophylactic drugs more affordable and improve the awareness of the disease and the information provided to travellers in the tropical health centres.

*Plasmodium falciparum *continues to be the most frequent species [9,10] in Barcelona as well as Spain in general [7], other European countries [17,18], and the USA [19,20]. However, *P. vivax *is prevalent in England and Australia [30] because the immigration in these countries is from Asia, where *P. vivax *is the most common species. The number of non-identified species (*Plasmodium sp*) can be explained in part by the inexperience of some centres and the high percentage of immigrants with very low parasitaemias, in which it is sometimes difficult to determine the species. Nevertheless, the non-identified species were clustered in the first few years of the study (Figure 4). The existence of *Plasmodium sp *could correspond to sub-clinical parasitaemia among semi immune immigrants from holoendemic malaria areas. Nowadays, the incorporation of a second generation of rapid diagnostic tests combining aldolase and histidine-rich protein II (HRP-2) and PCR would help to improve determination of the species.

The efficacy of chemoprophylaxis is a concern on a worldwide scale. Despite resistances, the different chemoprophylaxis guidelines could be effective [31], but there is a lack of awareness of the observed problem [32] and adherence is often inadequate [33], as seen in this study, particularly among immigrants since under 0.5% of VFR subjects followed recommendations. Compliance with chemoprophylaxis among those affected between 1989 and 2005 was lower than those affected between 1981 and 1987 [7], a phenomenon observed in other contexts as well [17-19]. Speculating 90% efficacy of chemoprophylaxis and 90% adherence, malaria could have been avoided in more than 294 of the 344 autochthonous cases.

Malaria in travellers is preventable if adequate measures are taken. The WHO calculates that between 1995 and 2015, the number of long distance trips will have doubled. In addition to the increase of risk of infection from more travel to endemic areas, the growth of resistance to medication should be considered, given the infrequent and poor use of chemoprophylaxis [34,35]. Some of the keys to the fight against this disease and its sometimes fatal consequences [36] include early diagnosis and treatment when a febrile syndrome is presented after a trip [37], more and better adherence to chemoprophylaxis [18,38], adjustment of drug doses such as primaquine to avoid relapses [39,40] and extending the use of repellents, impregnated clothes and mosquito nets [8,35]. Notification of cases in the public health system is also an essential tool in order to follow the tendencies of this important imported disease.

Some especially vulnerable groups will be exposed to the disease and its complications in the next few years, such as children, pregnant women [41], VFR (particularly in Spain where many of the immigrants from Africa are Equatorial Guineans), long term and elderly travellers [35,42,43]. The use of chemoprophylaxis and the characteristics of the most affected groups should be further researched and the aforementioned recommendations should be evaluated.

The high number of patients collected for this study was possible thanks to the collaboration during the years of the two centres of imported diseases and the epidemiology service of Public Health Agency of Barcelona. The joint work of the various health centres and public health services, as done in related diseases concerning immigration such as tuberculosis [44], is a course that should be followed. For the control and prevention of malaria, surveillance must improve in cities and developed countries because apart from better control, this could be a good reflection of the disease worldwide.

## Authors' contributions

JPM, PG, PCS, and JC designed the study, collected the data, analysed and prepared the first draft. All authors put forward different ideas, contributed to the interpretation of the data, early drafts and agreed the final draft.
